# Management of benign and suspicious breast lesions during the coronavirus disease pandemic: recommendations for triage and treatment

**DOI:** 10.6061/clinics/2020/e2097

**Published:** 2020-06-24

**Authors:** Flávia Abranches Corsetti Purcino, Carlos Alberto Ruiz, Isabel C.E. Sorpreso, Ana Maria Massad Costa, José Maria Soares-Júnior, Edmund Chada Baracat, José Roberto Filassi

**Affiliations:** I Setor de Mastologia, Disciplina de Ginecologia, Departamento de Obstetricia e Ginecologia, Hospital das Clinicas HCFMUSP, Faculdade de Medicina, Universidade de Sao Paulo, Sao Paulo, SP, BR; II Disciplina de Ginecologia, Departamento de Obstetricia e Ginecologia, Hospital das Clinicas HCFMUSP, Faculdade de Medicina, Universidade de Sao Paulo, Sao Paulo, SP, BR

## INTRODUCTION

The coronavirus disease (COVID-19) outbreak began in Wuhan, China, in December 2019; by March 11, 2020, it was declared a pandemic by the World Health Organization (WHO) (1). The response to COVID-19, caused by severe acute respiratory syndrome coronavirus 2 (SARS-CoV-2), has impacted health care routines worldwide. There has been a global demand for medical supplies to equip new intensive care units and improve personnel protection. Human and material resource allocation is also now a high priority for the maintenance of health care system function and the avoidance of mortality on a large scale. Guided by the WHO, countries have established measures to reduce the numbers of new infections. In the escalating phase of the disease, measures were taken to suspend non-urgent elective surgeries (2).

The first COVID-19 case in Brazil was officially reported on February 26, 2020; by June 1, 2020, approximately 500,000 cases and 30,000 deaths had been reported (3) and Brazil is the current epicenter of SARS-CoV-2 infection in Latin America. This scenario creates a challenge for the diagnosis and treatment of serious and prevalent diseases, including cancer. Before the COVID-19 outbreak, the Brazilian National Cancer Institute estimated approximately 66,000 new cases of breast cancer in 2020 (4). Benign breast diseases are also frequent and require health assistance at various levels of complexity (5). Therefore, a period of impaired access to early diagnosis of breast diseases during the COVID-19 pandemic is expected. In a recent publication, the Brazilian Society of Pathology and Brazilian Society of Surgical Oncology indicated a possible 70% reduction in the number of elective cancer surgeries in April and 50,000 missed cancer diagnoses in the country since the pandemic outbreak, corresponding to an expected 50% reduction in the detection of oncologic cases during this period (6).

A delay in the early diagnosis of breast cancer can worsen the outcomes (7). Delayed diagnosis is exacerbated by patient fear of SARS-CoV-2 infection, resulting in the postponement of appointments, examinations, and surgeries. Low-complexity care units also have reduced referrals because of discontinued propaedeutic activity during the pandemic. Fewer diagnoses results in reduced referral for treatment and subsequent deleterious outcomes in humans and on the economy. In the coming weeks or months, it may be necessary to manage an overwhelming influx of patients with breast disease or advanced breast cancer in a health care system that is already overwhelmed by COVID-19.

Postponed or re-scheduled diagnosis and treatment may be unavoidable in the escalating phase of COVID-19 infection (8). However, it is important to maintain or reschedule these vital services as soon as possible. Benign and suspicious breast lesions should be properly triaged and prioritized for treatment under the exceptional circumstances of the COVID-19 pandemic.

### Benign and suspicious breast lesion triage and treatment recommendations

In collaboration with surgical specialty societies, the American Society of Breast Cancer has made recommendations for prioritization based on a patient’s condition (9). Patients categorized as Priority A are in immediately life-threatening and clinically unstable conditions that require a prompt response, including evacuation of hematomas or drainage of breast abscesses. Patients categorized as Priority B are in non-critical conditions for which delays beyond 6-8 weeks could potentially impact the overall outcome. These include diagnostic imaging or Breast Imaging-Reporting and Data System (BI-RADS) category 4-5 screening mammograms, biopsies for abnormal mammograms, or breast symptoms and discordant biopsies likely to be malignant. Patients categorized as Priority C have stable conditions for which action can be delayed for the duration of the COVID-19 pandemic, including routine screening with mammogram, MRI, or breast ultrasound; excision of benign nodules; duct excision; follow-up of discordant biopsies likely to be benign; treatment of high-risk lesions/atypia/papilloma; or prophylactic surgery. This consortium also recommended the replacement of in-person visits with telemedicine when appropriate.

The recommendations of the American College of Surgeons are similar to those discussed above but also consider important aspects of pandemic phases and institutional resources that affect decision-making (10). In Phase I settings (semi-urgent), in which there are few COVID-19 patients, hospital resources and intensive care unit capacity are not exhausted, and the COVID-19 trajectory is not rapidly escalating, surgery should be restricted to patients likely to have compromised survivorship if the procedure is not performed within next 3 months. In the Phase I setting, discordant biopsies likely to be malignant should be clarified as soon as feasible and other breast surgeries, such as excision of benign lesions, duct excision, discordant biopsies likely to be benign, high-risk lesions (atypia, papilloma), and prophylactic surgery, should be deferred. The Phase II setting (urgent) is characterized by many COVID-19 patients, limited intensive care unit and ventilator capacity, limited supplies, or rapidly escalating COVID-19 trajectory within a hospital. Phase III is reached when all hospital resources are dedicated to COVID-19 patients, there is no ventilator or intensive care unit capacity, or supplies are exhausted. In Phases II and III, breast surgeries should be restricted to patients likely to have compromised survivorship if surgery is not performed within days or hours, respectively. All other breast procedures should be deferred.

The recommendations from the Society of Surgical Oncology also considered hospital resources (11). While individualization of each case is encouraged, there is clear guidance to perform abscess or infection surgeries after conservative management failure and to postpone other benign breast disease surgeries for at least 3 months.

The epidemiologic profile of COVID-19 in some countries has revealed poorer prognosis in the elderly. Age is a relevant aspect of the recommendations set forth by the European Society of Surgical Oncology (ESSO) (12). These guidelines state that patients over 70 years of age or elderly patients with comorbidities have higher risks of death from coronavirus and, when possible, should be seen after the pandemic is over. The ESSO also suggests no surgery for benign disease.

Some national specialty societies have also set forth their recommendations on the management of benign and suspicious breast lesions during the COVID-19 pandemic. The Brazilian Society of Surgical Oncology (13) considered the impact on prognosis when recommending surgeries during the COVID-19 pandemic. Delaying surgeries for some conditions, such as benign breast nodules, breast malformation, and gynecomastia, are considered to have no impact on prognosis. Postponing prophylactic surgeries and excision of atypical lesions has a very low impact on the prognosis. However, according to previous protocols, incision and drainage of breast abscess and hematoma evacuation are urgent procedures that should be performed as soon as feasible. New cases of breast disease should undergo triage by telemedicine if possible.

For symptomatic patients with palpable breast lesions, suspicious nipple discharge, breast pain, or an abscess without a prior oncologic diagnosis, the Brazilian Society of Mastology, the Brazilian College of Radiology, and the Brazilian Federation of Gynecology and Obstetrics Associations highlight the importance of estimating how clinically suspicious lesions are defined and how imaging information impacts decision-making, particularly in patients older than 60 years (14). These considerations should also be applied to the investigation of BI-RADS category 0, 4, and 5 findings, although percutaneous biopsy is generally indicated in the latter cases. Postponement of all screening methods is recommended despite a personal risk of breast cancer.

**Figure 1:**
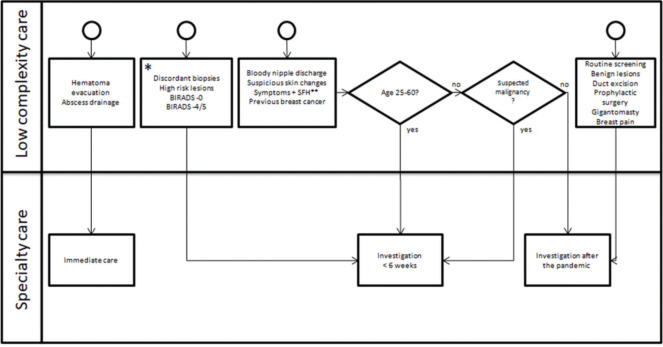
Proposed decision-making algorithm for the triage and treatment of benign and suspicious breast lesions during the coronavirus disease 2019 (COVID-19) pandemic. *If there is a high incidence of COVID-19 and exhaustion of resources, consider postponing the investigation of discordant biopsies likely to be benign. Chemoprophylaxis is a substitute for surgery in high-risk lesions. **SFH: strong family history (two or more first-degree relatives affected). Adapted from The American Society of Breast Surgeons (9).

These recommendations provide a detailed guide for caregivers regarding decision-making and are intended to provide the best balance between morbidity and mortality from breast disease, risk of self and healthcare team exposure to COVID-19, and consumption of health resources. With a goal of guiding service flow, triage, and treatment of benign and suspicious breast lesions in low-complexity and specialty care settings, we proposed recommendations as outlined in the flowchart in Figure 1. An important characteristic considered in the development of these guidelines was heterogeneity in COVID-19 burden among different regions in Brazil as highly impacted areas contrast with spared regions.

These recommendations regarding the users of the public health system are predicated on the free access and availability of safe health care facilities to mitigate risks for patients and professionals (15). Clinical and surgical outpatient procedures are preferred. Special efforts to provide easy access to radiology examinations and pathology results are of value. Telemedicine should be used to allow safe and virtual contact with new and established patients when possible (16). The use of telemedicine during the COVID-19 pandemic was recently recognized as feasible and ethical by the Federal Council of Medicine in Brazil (17); however, the lack of ability to conduct physical examinations through this modality can be a limitation. Nevertheless, local authorities’ recommendations on social distancing should be respected.

## CONCLUSIONS

Health routines and surgical algorithms are currently being reviewed and updated worldwide owing to the COVID-19 pandemic. Meanwhile, early breast disease diagnosis remains a priority in the Brazilian public health system (18). Here, we intended to guide and simplify the triage and treatment of benign and suspicious breast lesions during this pandemic. Although these are days of great uncertainty, it is vital to maintain breast disease awareness and guarantee a safe health infrastructure for patients and healthcare workers.

## AUTHOR CONTRIBUTIONS

Purcino FAC, Ruiz CA and Costa AMM were responsible for the manuscript drafting, writing, editing and review. Baracat EC and Filassi JR were responsible for study supervision and manuscript drafting, writing, editing and review. Sorpreso ICE and Soares-Júnior JM were responsible for the study conceptualization and supervision, as well as manuscript drafting, writing, editing and review.
